# Sex-based differences in the activation of peripheral blood monocytes in early Parkinson disease

**DOI:** 10.1038/s41531-021-00180-z

**Published:** 2021-04-13

**Authors:** Samantha M. Carlisle, Hongwei Qin, R. Curtis Hendrickson, Jordana E. Muwanguzi, Elliot J. Lefkowitz, Richard E. Kennedy, Zhaoqi Yan, Talene A. Yacoubian, Etty N. Benveniste, Andrew B. West, Ashley S. Harms, David G. Standaert

**Affiliations:** 1 Alabama Morris K. Udall Center of Excellence in Parkinson’s Disease Research, University of Alabama at Birmingham, 1719 Sixth Ave. South, Birmingham, AL USA; 2 Center for Clinical and Translational Science, University of Alabama at Birmingham, Birmingham, AL USA; 3 Department of Chemistry and Biochemistry, New Mexico State University, Las Cruces, NM USA; 4 Department of Cell, Developmental and Integrative Biology, University of Alabama at Birmingham, Birmingham, AL USA; 5 Department of Neurology, University of Alabama at Birmingham, Birmingham, AL USA; 6 Department of Pharmacology & Cancer Biology, Duke University, Durham, NC USA

**Keywords:** Parkinson's disease, Parkinson's disease

## Abstract

Increasing evidence supports the role of brain and systemic inflammation in the etiology of Parkinson disease (PD). We used gene expression profiling to examine the activation state of peripheral blood monocytes in 18 patients with early, untreated PD and 16 healthy control (HC) subjects. Monocytes were isolated by negative selection, and gene expression studied by RNA-seq and gene set enrichment analysis. A computational model that incorporated case/control status, sex, and the interaction between case/control status and sex was utilized. We found that there was a striking effect of sex on monocyte gene expression. There was inflammatory activation of monocytes in females with PD, with enrichment of gene sets associated with interferon gamma stimulation. In males, the activation patterns were more heterogeneous. These data point to the importance of systemic monocyte activation in PD, and the importance of studies which examine the differential effects of sex on pathophysiology of the disease.

## Introduction

Recent studies have revealed a critical role for activation of the immune system in the pathogenesis of Parkinson disease (PD)^1^. There is clear evidence for neuro-inflammation within the brain and increasing evidence that there is also abnormal activation of circulating immune cells. In the brain, there is activation of microglia (the intrinsic myeloid cells of the brain) and macrophages^2^, increased production of cytokines^3^, and infiltration of CD4 + and CD8 + T-cells^4^. In the circulation, there is an increase in inflammatory cytokines including IL-6 and IFN- γ^5^, modifications of circulating monocytes^6,7^, changes in regulatory and effector T-cells^8^, and induction of memory T-cells with a specific response to alpha-synuclein (α-syn)^9,10^, a protein with a central role in the pathophysiology of PD. In animal model systems, blockade of these inflammatory responses can attenuate disease progression, pointing the way to a novel approach to neuroprotective treatments for human PD^11–14^.

Blood monocytes appear to have a central role in immune signaling in PD^15^. Monocytes are cells of myeloid lineage, derived from the bone marrow. They are phagocytic and play a critical role in immune surveillance through engulfing, processing, and presenting foreign antigens for recognition by the adaptive arm of the immune system. Evidence linking blood monocytes to the pathogenesis of PD includes gene expression studies, which show prominent expression of PD-associated genes in monocytes^16^; prior human studies, which have demonstrated evidence for alterations of blood monocytes in PD^6^; and work in animal models, which has shown that entry of monocytes from the blood into the brain is a critical step in α-syn mediated neurodegeneration^14^. Blood monocytes which enter the brain can have a morphology that is nearly identical to microglia, but microglia have a distinct embryological origin in the yolk sac, and different properties when activated^17,18^.

Monocytes infiltrate into target tissues in response to chemokines and differentiate into activated states. While these states are often referred to as M1, or “pro-inflammatory” and M2, “anti-inflammatory,” this is a great oversimplification of the biology of these cells which, in fact, can assume a large number of different activation states when driven by different soluble and environmental factors^19^. In disorders including traumatic brain injury and multiple sclerosis, and animal models of these conditions, the balance between pro- and anti-inflammatory activation has been shown to have a critical role; promoting M2-like phenotypes can reduce injury and promote recovery^20–22^. In PD, there is evidence for a diversity of monocyte activation states, but little available data on the details of monocyte phenotypes^23^.

Here, we have used molecular approaches to examine the state of activation of peripheral blood monocytes in human PD. In contrast to prior studies, which examined monocytes in heterogenous cohorts of patients, we have recruited a population of patients with early, untreated PD. To isolate blood monocytes, we utilized a negative selection approach, which removes other cell types while leaving the monocytes unaffected by the separation method. We analyzed the monocytes, using both conventional flow cytometry, as well as analysis of bulk RNA expression. Bioinformatic approaches were used to explore the spectrum of activation signatures present in these cells. We found evidence for inflammatory activation of blood monocytes in PD, with an unexpectedly strong effect of sex on the phenotype of monocyte activation.

## Results

### Patient population

Subjects with PD (UK Brain Bank criteria) within 2 years of symptom onset and not taking any anti-parkinsonian medication were recruited, along with age-matched healthy control (HC) subjects. Detailed inclusion and exclusion criteria are described in Methods. A total of 21 male (10 HC, 11 PD) and 13 female (6 HC, 7 PD) subjects were enrolled. Descriptive statistics for the patient subject cohort are presented in Table 1. Evidence of a difference between PD and HC in the proportion of males, proportion of Caucasian race, and age of patients (at blood collection visit) were not observed. However, family history of PD, Movement Disorder Society-Unified Parkinson’s Disease Rating Scale (MDS-UPDRS; I-III & total) scores, Montreal Cognitive Assessment (MoCA) score, and Parkinson’s Disease Questionairre-39 (PDQ39) score were significantly different between cases and HC (*p* < 0.05 for all). Of note is that no patient within the cohort scored any points on the MDS-UPDRS IV, a scale measuring symptoms of later stage PD, therefore significance was not tested. Additionally, there were no significant differences in any of the subject cohort characteristics, including age, between males and females (data not shown).

**Table 1:** Cohort characteristics.

Variable	Cases, PD (*n* = 18)	Controls, HC (*n* = 16)	*p*-value
Males, *n* (%)	11 (61.1%)	10 (62.5%)	1.00
Age at Visit (mean ± SD) years	61.1 ± 10.5	65.4 ± 9.7	0.21
Caucasian race, *n* (%)	18 (100%)	15 (93.8%)	0.47
Family History of PD, *n* (%)	5 (27.8%)	0 (0%)	0.046
MDS UPDRS I (median [Q1, Q3])	8.5 [3.2, 10.8]	3 [1, 4.2]	0.01
MDS UPDRS II (median [Q1, Q3])	6.5 [3.2, 9.8]	0 [0, 0]	<0.0001
MDS UPDRS III (median [Q1, Q3])	21.5 [13.2, 33.8]	0 [0, 0]	<0.0001
MDS UPDRS IV (median [Q1, Q3])	0 [0, 0]	0 [0, 0]	NA_1_
MDS UPDRS total (median [Q1, Q3])	34 [26.2, 48.8]	3 [1, 5]	<0.0001
MoCA total (median [Q1, Q3])	25 [24, 26.8]	26.5 [26, 28]	0.04
PDQ39 total (median [Q1, Q3])	72 [42.5, 111]	25 [18.5, 38.5]	0.003

^1^MDS-UPDRS IV score was not tested for significance as all subjects had a score of 0.

### Monocyte isolation and characterization

**Fig. 1:**
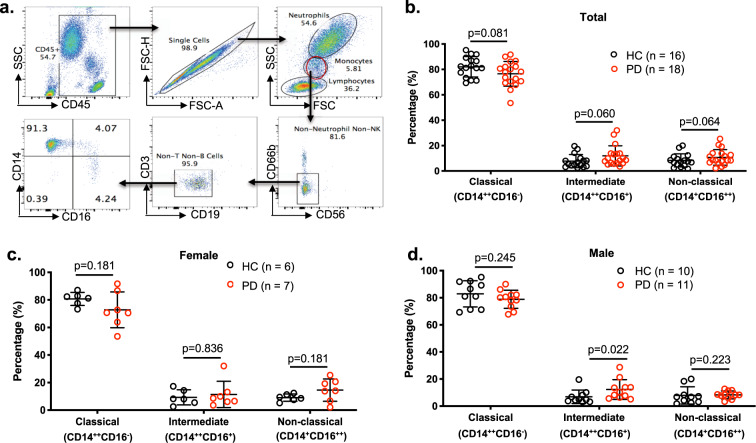
Gating strategy for monocyte subsets and the analysis of monocyte subsets in PD patients and healthy controls. **a** Single CD45^+^ white blood cells (WBCs) were gated as lymphocytes, monocytes, and neutrophils. The monocyte populations were further gated as CD56^−^CD66b^−^CD3^−^CD19^−^ cells, and then monocyte population was classified as Classical (CD14^++^CD16^−^); Intermediate (CD14^++^CD16^+^); and Non-classical (CD14^+^CD16^++^) monocyte subsets. Classical, Intermediate, and Non-classical monocytes from Parkinson disease (PD) and healthy control (HC) subjects were analyzed in total (**b**), female (**c**), and male (**d**) subjects. Statistical analysis was done using Student’s t-test with one-tailed distribution and two-sample equal variance (homoscedastic). Frequencies of cell subsets are shown as mean ± SD. Black dots represent HC subjects and red dots represent PD subjects.

Monocytes from de novo PD subjects (*n* = 18) and HC subjects (*n* = 16) were analyzed to examine immune profiles (Fig. 1). PBMCs were stained with antibodies to CD45, CD3, CD14, CD16, CD19, CD56, and CD66b, and then single CD45^+^ live cells were gated as CD56^−^CD66b^−^CD3^−^CD19^−^ monocytes using flow cytometry analysis (Fig. 1a). As the next step, monocyte subsets were determined using CD14 and CD16, evaluating Classical (CD14^++^CD16^−^), Intermediate (CD14^++^CD16^+^), and Non-classical (CD14^+^CD16^++^) monocytes (Fig. 1a). Our results demonstrated moderate evidence of a decrease in the population of CD14^++^CD16^−^ Classical monocytes and moderate evidence of an increase in both Intermediate (CD14^++^CD16^+^) and Non-classical (CD14^+^CD16^++^) monocytes in PD compared to HC subjects (Fig. 1b). However, no significant differences in naïve B-cells, non-switched memory (NSM) B-cells, and double-negative (DN) B-cells were observed either in male or female PD subjects (data not shown). Convincing evidence of a difference in Classical, Intermediate, and Non-classical monocytes between PD and HC subjects was not observed in females (Fig. 1c). In males, we did not observe convincing evidence of a difference in Classical and Non-classical monocytes, however, we did observe a difference in Intermediate monocytes between PD and HC (Fig. 1d). Additionally, we did not observe a case/control interaction with male/female, therefore the observed marginal differences between PD and HC does not depend on sex.

### RNA expression analysis

RNA-seq was performed on CD14 + monocytes isolated from each of the individual subjects in the study, a total of 34 samples. All but two sequencing samples had alignment rates greater than 90% for reads uniquely mapped to the human hg38 reference genome; the alignment rate of reads uniquely mapped to the human hg38 reference genome for samples PDJH981CJA and PDDP165AWY were ~87% and ~88%, respectively, with a slightly greater percentage (~9%) of reads mapping to multiple loci in both samples compared to the other sequencing samples. All samples had at least 22.8 million reads aligned to the human hg38 reference genome. No other sequencing quality metrics were found to be problematic for any samples.

**Fig. 2:**
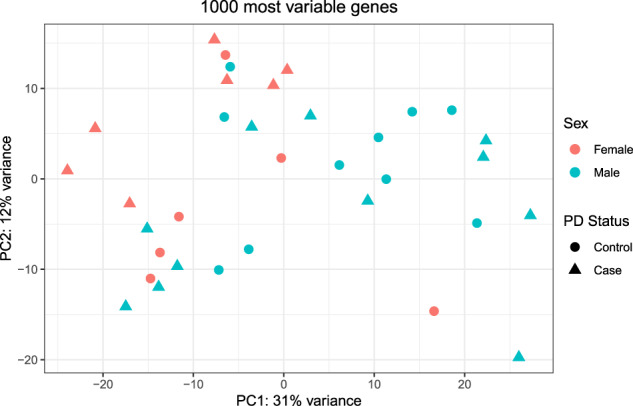
Principal component analysis reveals primary effect of sex on gene expression. Principal Component Analysis (PCA) of the 1000 most variably expressed genes in monocytes from our RNA-seq dataset was conducted and the first two principal components were plotted. A primary effect of sex on gene expression was observed. Circles represent healthy control subjects (*n* = 16) and triangles represent PD subjects (*n* = 18); females (*n* = 13) are filled orange and males (*n* = 21) are filled light blue.

Principal component analysis (PCA) was used to examine the unbiased grouping of PD and HC samples utilizing the 1000 most variable genes in the entire cohort of 34 subjects (Fig. 2). Surprisingly, the first two principal components revealed by this analysis separated the subjects by sex but not the disease state. While some of these genes, such as *XIST* and *RPS4Y1*, are known to be sex-linked and thus were expected, the effect of sex on monocyte gene expression was much broader than just those genes known to be associated with sex. Based on this finding, a model that incorporated case/control status, sex, and the interaction between case/control status and sex was utilized for determining differential expression between PD and HC subjects; age was not included as a factor in the model as there was no statistical difference in age between cases and controls (*p* = 0.21) or males and females (*p* = 0.77; data not shown). Subsequent analyses focused on differential expression between PD and HC separately in male and female subjects.

**Fig. 3:**
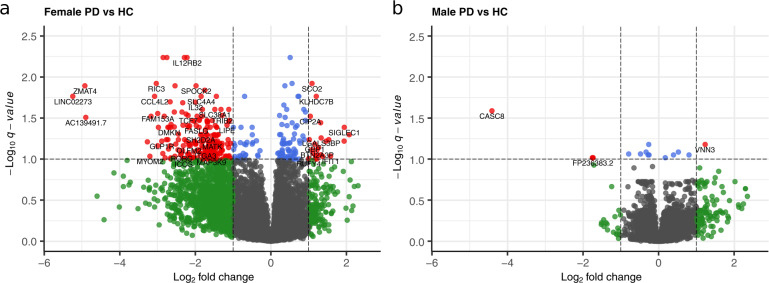
More genes were differentially expressed between PD and HC in females than males. Differential expression of genes between Parkinson disease (PD) and healthy control (HC) subjects were visualized with volcano plots in females (**a**) and males (**b**). A *q*-value threshold of 0.1 and a fold-change (FC) threshold of 2 (log2 FC of 1) was applied to visualization. Each individual dot represents a single gene; black dots represent genes with a non-significant *q*-value and |log2 FC | < 1, green dots represent genes with a non-significant *q*-value and |log2 FC | > 1, blue dots represent genes with a significant *q*-value and |log2 FC | < 1, and red dots represent genes with a significant *q*-value and |log2 FC | > 1. Select genes are labeled in each plot. Note that while *CCL4* is both significant and has a large fold change (*q*-value < 0.0001, FC = −3.7) in the females, it is located outside the plotting region of the female volcano plot.

**Fig. 4:**
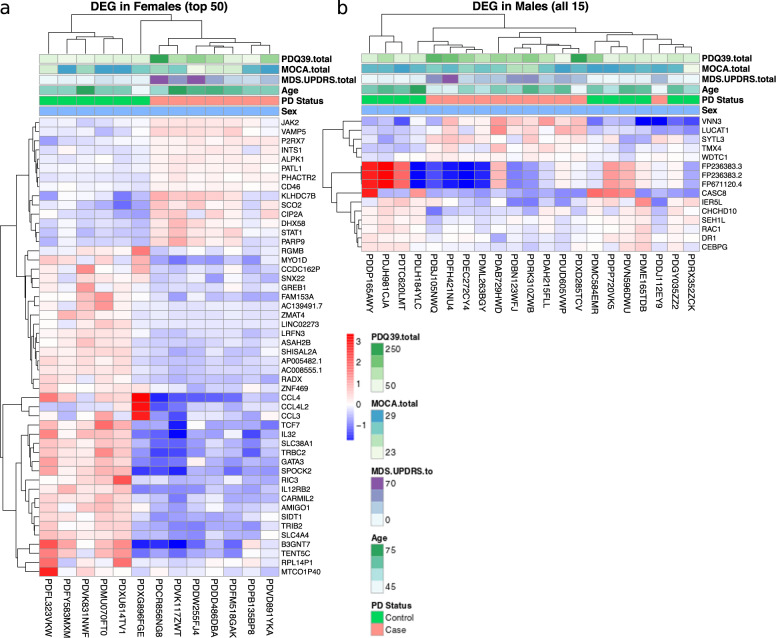
Hierarchical clustering reveals differentially expressed genes between PD and HC subjects are more pronounced in females than males. Hierarchical clustering was used to examine the unbiased grouping of Parkinson disease (PD) and healthy control (HC) subject samples utilizing the 50 most significant DEG in females (**a**), or all DEG in males (**b**). In females, there was clear separation by PD vs. HC, while the gene expression data in the males was more heterogenous and did not cluster samples into two clear groups. Each column represents an individual subject while each row represents a single gene. Normalized gene expression values are plotted. Additionally, each subject’s score on the Parkinson’s Disease Questionaire-39 (PDQ39), Montreal Cognitive Assessment (MoCA), total Movement Disorder Society-Unified Parkinson’s Disease Rating Scale (MDS-UPDRS), their age, and their Parkinson disease status is plotted above the gene expression values.

In females, 347 statistically significant differentially expressed genes (*q* < 0.10) between PD and HC were observed (Fig. 3a). Overall, there was clear separation by disease status in the differentially expressed genes in the female cohort (Fig. 4a). In the top 50 differentially expressed genes between PD and HC, there were two clear groups of genes, distinguished by increased gene expression in cases and decreased gene expression in HC in the first group of genes, and the reverse for the second group of genes.

In males, only 15 statistically significant differentially expressed genes (*q* < 0.10) between PD and HC subjects were observed (Fig. 3b). This is despite the larger sample size for males (21 males vs. 13 females), and likely reflects greater heterogeneity in the male population (Fig. 4b). With this small number, there was limited overlap between the differentially expressed genes in males and females. There was only a single differentially expressed gene in common between cases and HC in the female and male cohorts, that being *CEBPG*, a widely expressed transcription factor. Further, *CEBPG* was increased in HC males (compared to PD) but increased in PD females (compared to HC) however the fold change between PD and HC was quite small in both. This lack of overlap in differences in gene expression between cases and controls in males and females suggests that either the mechanisms of the pathogenic process, or the systemic response to these processes, differ between males and females.

### Validation of a subset of significant genes via qRT-PCR

**Fig. 5:**
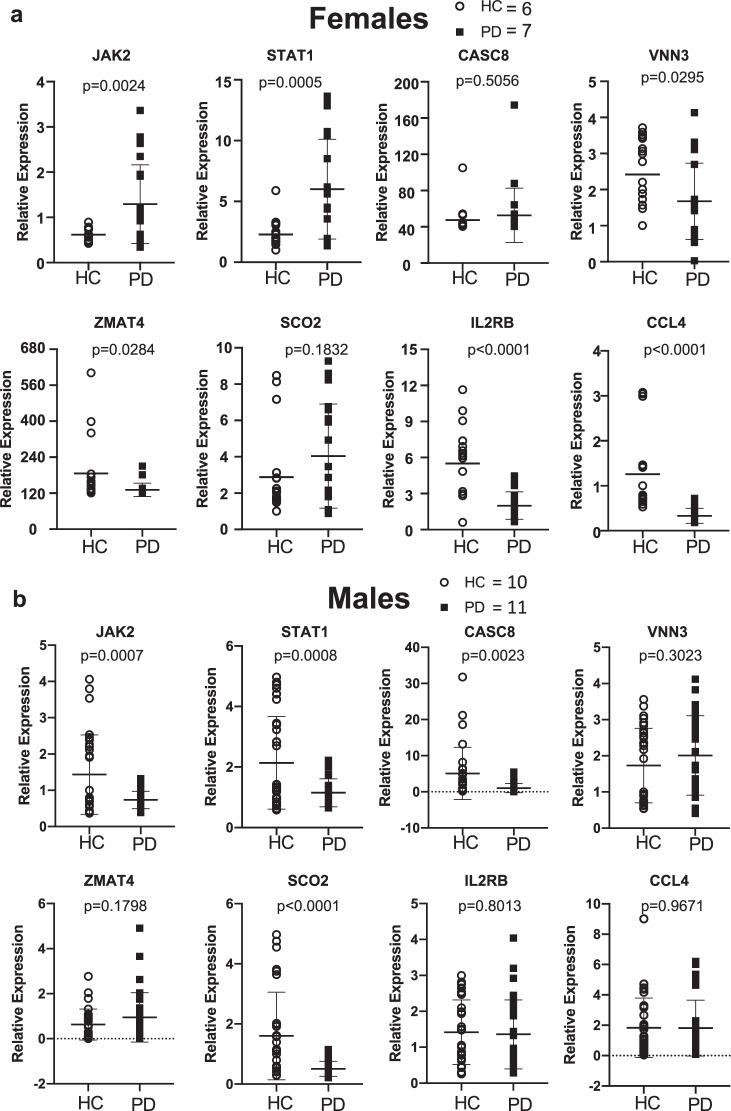
mRNA expression in monocytes determined by qRT-PCR in HC and PD patients. RNA was extracted from isolated monocytes and qRT-PCR performed using primers for *JAK2*, *STAT1*, *CASC8*, *VNN3*, *ZMAT4*, *SCO2*, *IL2RB*, *CCL4* with three repeated experiments in (**a**) females; HC = 6 and PD = 7 and (**b**) males; HC = 10 and PD = 11. Statistical significance was assessed with Student’s t-tests. Relative gene expression is shown as mean ± SD and all individual replicates are plotted.

qRT-PCR was conducted on all samples for the following genes: *JAK2*, *STAT1*, *CASC8*, *VNN3*, *ZMAT4*, *SCO2*, *IL2RB*, and *CCL4* to validate to RNA-seq results. Relative gene expression of each gene was compared between HC and PD subjects in each sex separately (Fig. 5). In general, there was close correspondence between the RNA-seq observations and the qRT-PCR measurements. In the RNA-seq data, *JAK2*, *STAT1*, and *SCO2* were significantly increased in PD compared to HC in females. qRT-PCR reproduced this observation for *JAK2* and *STAT1*; *SCO2* trended in the expected direction but did not achieve statistical significance. Additionally, *CCL4* and *ZMAT4* were significantly decreased in the RNA-seq data; these observations were replicated in the qRT-PCR data. In the RNA-seq data, *VNN3* was significantly increased while *CASC8* was significantly decreased in PD compared to HC. The qPCR data validated this observation for *CASC8*; while the difference between *VNN3* in PD and HC was not significant the trend was directionally consistent. Interestingly, while *JAK2* and *STAT1* were not found to significantly differ between PD and HC in males in the RNA-seq data, the qPCR showed both to be significantly decreased in PD compared to HC in males. Of course, it is important to note that the absence of a significant *q*-value in the RNA-seq or *p*-value in the qPCR data can not be interpreted to mean there is no difference between the two groups; a difference might be detected with a larger sample size.

### Pathways affected by PD in monocytes

**Fig. 6:**
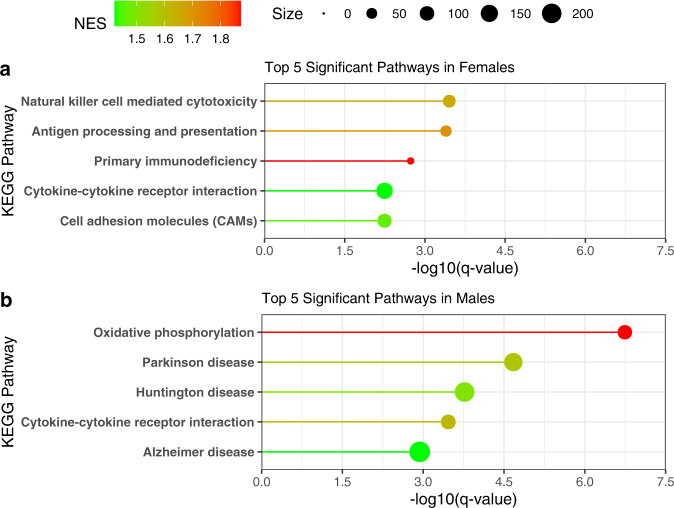
Immune pathways are disrupted between PD and HC in both sexes. Pathway analysis was conducted using Gene Set Enrichment Analysis (GSEA) and the human Kyoto Encyclopedia of Genes and Genomes (KEGG) pathways in females (**a**) and males (**b**), separately. Genes ranked by the absolute value of the Wald test statistic generated by differential expression analysis between PD and HC were utilized in the analysis. The KEGG pathway name is labeled on the y-axis, the -log10 of the *q*-value is plotted on the x-axis, the line and dot plotted are colored by the normalized enrichment score (NES) with the highest NES plotted in red and the lowest (from the significant pathways plotted) in green. The size of the dot in the figure represents the number of genes from our input that were in that specific pathway.

Pathway analysis was conducted by comparing enrichment of differentially expressed genes between PD and HC subjects to the human KEGG pathways via Gene Set Enrichment Analysis (GSEA) (Fig. 6). Pathway analysis in females revealed a consistent signature of alterations in inflammatory signaling, with the top 5 KEGG pathways including the natural killer cell-mediated cytotoxicity pathway, antigen processing, and presentation pathway, primary immunodeficiency, and cytokine-cytokine receptor interaction pathway. As in the single gene analysis, pathway analysis in males revealed a more heterogenous pattern: the KEGG cytokine-cytokine receptor interaction pathway was altered, in common with females, but other pathways altered in males were more diverse and associated primarily with disease state groupings rather than specific mechanisms (Fig. 6b).

### Prediction of monocyte activation states

We also used GSEA to examine the state of activation of monocytes in our patient cohort. The reference dataset for this was the study by Xue et al.^24^, which used diverse activation signals to differentiate human blood-derived macrophages and identify modules of genes, using Weighted Gene Co-Expression Network Analysis (WGCNA), that were both shared and stimulation-specific, transcriptional regulators and activation programs. We assessed the degree of enrichment of differentially expressed genes between PD and HC to these modules (gene sets) to predict the activation states differentially present in our cohort.

**Fig. 7:**
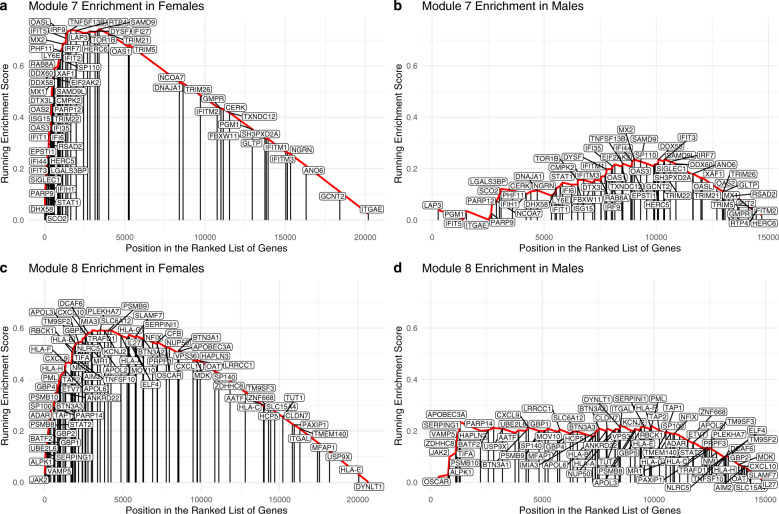
Classical M1 pro-inflammatory gene modules enriched for differences between PD and HC in female cohort but not male cohort. Gene Set Enrichment Analysis (GSEA) was performed separately for each sex. Enrichment results for Module 7 in females (**a**), enrichment results for Module 7 in males (**b**), enrichment for Module 8 in females (**c**), and enrichment results for Module 8 in males (**d**) are shown. Two gene modules (of the 49 tested) were enriched in females (Modules 7 and 8, FDR *q*-value < 0.001). Both are classical “M1” pro-inflammatory gene programs, and strongly associated with response to IFNγ. No gene modules achieved statistical significance in males. Genes in each individual module are labeled at the position in which they appear in the ranked gene list (ranked by the absolute value of the Wald test statistic value between PD and HC) for females and males.

In females, we detected enrichment of 2 different gene modules, Modules 7 and 8, with FDR *q*-values < 0.001 (Table 2, Fig. 7a, b). Both of these modules are classical “M1” pro-inflammatory gene programs, and strongly associated with response to interferon-γ. In the males, none of the gene modules achieved statistical significance (Fig. 7c).

**Table 2:** Monocyte activation state modules significantly enriched between PD vs HC subjects in females.

Gene modules from Xue et al. enriched in females	Module associations with stimulators from Xue et al.				GSEA normalized enrichment score	*p*-value	FDR *q*-value
	Classical	IFNg_2_	IL-4_2_	TPP_2_			
Module 7_1_	M1	0.26	−0.15	−0.31	2.10	1.00E-05	<0.001
Module 8_1_	M1	0.61	0.24	−0.16	1.70	1.00E-05	<0.001

^1^Module name was taken directly from Xue et al’s work and refers to the same module of genes reported in their work.

^2^Pearson correlation coefficients of the eigengene for that module and the specific stimulation condition from the original Xue et al. work.

## Discussion

In this study, we have found evidence for differential patterns of gene expression in blood monocytes from patients with early PD. Although prior studies have described differences in blood monocytes between PD patients and controls, most of these studies have included patients with a wide range of disease duration and severity, and who are receiving a number of different treatments^6,25^. Here, we have examined a group of subjects recruited at the earliest stage at which PD can be diagnosed, removing some of the sources of heterogeneity in the previous studies. The presence of differences in monocyte gene expression at this early stage of the disease supports the idea that systemic immune system activation is a feature of early PD.

Differences in gene expression based on sex, especially in an immune context, are well known^26^ and have been described previously in blood monocytes as well as in a variety of different tissues^27^, but much less is known about the interactions between PD and sexually dimorphic patterns of gene expression. In examining the specific genes which are differentially expressed by monocytes in PD vs. HC, we observed a strong effect of sex on monocyte gene expression in both PD and HC populations. These sexually dimorphic features are evident at the level of PCA, which shows separation between males and females in the overall patterns of monocyte gene expression (Fig. 2). When the data are separated by sex, there is a clear signature of differential expression between female PD and female HC subjects (Figs. 3 and 4). In males, the effect differences between PD and HC monocytes are fewer, and there appears to be greater heterogeneity in the samples. In an earlier study of brain dopamine neurons from human substantia nigra, we also observed both effects of sex on neuronal gene expression and interaction between sex and the effects of PD on neurons^28^. Some specific sexually dimorphic genes, such as the male gene SRY, have been directly linked to the susceptibility of dopamine neurons to injury^29^. There are also important sex-based differences in the phenotype of PD, most notably the greater frequency of the disease in males than females, but there is also evidence for differences in frequency of tremor and imbalance, response to medications, and cognitive features of PD^30^.

We validated our results within the study cohort using qRT-PCR, but validation in additional, independent cohorts is an important next step. Unfortunately, few prior studies of inflammation in PD have considered sex effects or included sufficient numbers of men and women in order to detect these effects. Our results inform the design of future studies, by emphasizing the critical importance of inclusive enrollment of both sexes in studies of PD, and the need to consider that there may be sex-based differences in the underlying mechanism of or response to the disease process.

Duration of disease is also an important consideration. We have focused here on early, untreated PD, the earliest stage at which clinical PD can be diagnosed. Evidence is accumulating that the cytokine inflammatory signature of PD may be most intense early, just after clinical diagnosis, and may wane in more advanced stages^5,31^. We have recently reported that the same trend may be present with respect to T cells specific for α-syn, which seem to be most abundant shortly after diagnosis, and decline in subsequent years^10^. Of course, the presence of an inflammatory state in a disease does not establish causation; while it is possible that ongoing inflammation is etiological, there is also the possibility that some immune changes are in fact reactive to the disease process. This question has driven a lot of interest in the biological processes associated with the “prodromal” state of PD that may precede the clinical diagnosis^32^. Little is known about inflammatory signals in this state, although we have observed T cell responses years prior to diagnosis in a single case, and there has been some study of inflammation in REM Behavior Disorder, which is viewed as a pre-motor stage of PD^33^. It would be of great importance to study monocyte activation in men and women with prodromal PD.

Monocytes can be activated by a variety of different stimuli, and these lead to activated cells with a range of different activities along a spectrum of “pro-” and “anti-” inflammatory states. To examine the activation state of monocytes in our patient population, we used GSEA and employed reference gene sets described by Xue et al.^24^ These investigators used human blood-derived macrophages and applied a total 28 different stimuli. Gene expression analysis was conducted after stimulation and was used to identify a set of 49 distinct co-expression modules which are differentially activated by different stimuli. These modules were then used as gene sets in GSEA of differentially expressed genes from our monocyte RNA-seq data. Using this approach, GSEA revealed that in females, there is strong activation of blood monocytes with enrichment of two specific modules, both of which are strongly stimulated by interferon gamma. This is the hallmark of the classical pro-inflammatory “M1” phenotype. Monocytes of this kind are typically associated with pro-inflammatory actions that may lead to tissue injury. In contrast, we did not observe any statistically significant enrichment of monocyte activation modules in males with early PD compared to HC subjects. It is possible that this reflects a more mixed response in males, with both pro- and anti-inflammatory changes.

A limitation of this research is that we have studied pooled monocytes from individual subjects rather than utilizing single-cell sequencing approaches, and thus we are looking at responses averaged over a large number of monocytes. Single-cell approaches would be a logical next step and are likely to reveal the heterogeneity of the monocyte response in PD. Our flow cytometry analysis suggests the possibility of differential pathways activated in CD16-hi versus CD16-lo populations of monocytes in early PD that would be important to understand on a single-cell level.

Identification of an inflammatory signature in the blood of PD patients opens the door to novel approaches to both biomarkers and treatment. As a biomarker, assessment of monocyte activation has potential as a measure of disease activation and possibly the on-target efficacy of immunomodulatory treatment. As a target of therapy, we have previously shown that in animal models, blocking entry of blood monocytes into the brain prevents α-syn driven neurodegeneration^14^. A similar strategy, such as targeting CCL2 or one of the other chemokines that are critical for monocyte entry into organs, might be effective in human PD.

## Methods

### Ethics

This study was reviewed and approved by the University of Alabama at Birmingham (UAB) Institutional Review Board. All subjects signed written informed consent to participate in this study.

### Patient population

Patients with early PD were diagnosed using UK Brain Bank criteria (bradykinesia and either: 4–6 Hz resting tremor or rigidity). We included male or female subjects that were within 2 years of diagnosis, who were age 30 years or older at time of PD diagnosis, and who were Hoehn and Yahr stage I-II at the time of study entry. All PD subjects had no history of prior treatment with PD medications. Other exclusion criteria for PD subjects included clinical suspicions of atypical PD syndromes. Healthy controls were over 30 years old, had no current diagnosis of PD or other neurodegenerative disorder, no history of PD in first-degree blood relatives, and scored positive on no more than three items on the PD Screening Questionnaire^34^.

**Fig. 8:**
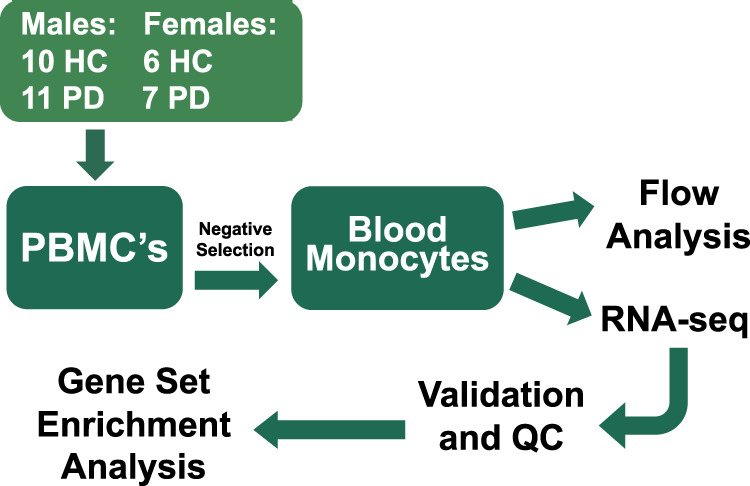
Overview of methods. Peripheral blood mononuclear cells (PBMCs) from healthy control (HC) subjects and Parkinson Disease (PD) subjects were collected. Monocytes were then isolated using negative selection. Flow analysis was conducted, and RNA was isolated for RNA-seq analysis and qRT-PCR. The resulting data were analyzed for differences between PD and HC subjects.

Degree of PD burden was assessed using MDS-UPDRS, MoCA, and PDQ39. Family history of PD, defined as a grandparent, parent, or sibling diagnosed with PD, was also evaluated for each subject enrolled. The original recruited and enrolled cohort contained 40 subjects, however six were excluded because they failed quality control (either insufficient RNA for sequencing, or RNA subject ID did not match DNA subject ID). Therefore, a total of 34 subjects, 21 male (11 PD, 10 HC) and 13 female (7 PD, 6 HC) subjects were included in the subsequent analysis (Fig. 8). All subjects signed written informed consent to participate in this study.

### Sample collection

Up to 50 mL of venous blood was collected using standard venipuncture into tubes containing EDTA from each PD and HC subject. All samples were processed within 2 h of collection. Peripheral blood mononuclear cells (PBMCs) were then isolated from blood by low-density gradient centrifugation using lymphocyte separation medium (Ficoll-PaqueTM, 25-072-CV), as described^10^ From ~50 mL of blood, we obtained ~40–60 × 106 PBMCs (Fig. 1). Next, monocytes were isolated using the EasySepTM Human Monocyte Isolation Kit (Catalog #19359 C), which isolates highly purified CD14 + monocytes by immunomagnetic negative selection from 20 × 106 PBMCs. Unused PBMCs were aliquoted at 10 × 106/vial and cryopreserved in liquid nitrogen. Two million monocytes were obtained, 0.5 × 106 cells were subjected to flow cytometry analysis.

### Flow cytometry analysis for monocyte subsets

PBMCs for phenotyping were blocked with 2% mouse serum prior to labeling with fluorochrome-conjugated monoclonal antibodies to surface markers of lymphocytes, monocytes, and neutrophils, CD3, CD14, CD16, CD19, CD56, and CD66b (Biolegend, San Diego, CA). Cells were fixed with 2% paraformaldehyde. Flow cytometry was performed on an LSRII flow cytometer (BD Biosciences, San Jose, CA), and data were analyzed using FlowJo software (Tree Star, Inc, Ashland, OR)^35,36^. Isotype-matched mouse monoclonal antibodies were used where appropriate to exclude non-specific antibody binding. CD45^+^CD3^−^CD19^−^CD56^−^CD66b^−^ monocytes were gated using forward scatter and side scatter, and single cells were identified using forward-scatter area versus width. Monocyte subsets were identified as Classical (CD14^++^CD16^−^); Intermediate (CD14^++^CD16^+^); and Non-classical (CD14^+^CD16^++^) monocyte subsets. The primary statistical analyses were done by comparing percentages of monocyte subsets in CD45^+^CD3^−^CD19^−^CD56^−^CD66b^−^ monocytes using relevant markers in PD patients versus HC by Wilcoxon’s test. Frequencies of cell subsets are shown as mean ± SD.

### RNA isolation and quantitative RT-PCR

Total RNA for each individual sample was extracted using Trizol from approximately 1.5 × 106 patient monocytes (all RIN values >9) in-house following manufacturer’s instructions. For quantitative RT-PCR (qRT-PCR) analysis, 1,000 ng RNA was reverse-transcribed into cDNA using M-MLV Reverse Transcriptase (Promega) as described^37^. cDNA was subjected to qRT-PCR using TaqMan primers (Thermo Fisher Scientific; *JAK2*: Hs01078136_m1, *STAT1*: Hs01013996_m1, *CASC8*: Hs01908214_s1, *VNN3*: Hs01125168_m1, *ZMAT4*: Hs00226442_m1, *SCO2*: Hs04187024_m1, *IL2RB*: Hs01081697_m1, and *CCL4*: Hs99999148_m1). Relative gene expression was calculated according to the ΔΔ threshold-cycle (Ct) method using the housekeeping gene 18 s rRNA as the control. Statistical significance was assessed with Student’s t-tests.

### RNA-seq Library-prep

Extracted RNA was sent to GENEWIZ, LLC (South Plainfield, NJ, USA) for library preparation and sequencing. RNA samples were quantified using Qubit 2.0 Fluorometer (Life Technologies, Carlsbad, CA, USA) and RNA integrity was checked with 2100 Bioanalyzer (Agilent Technologies, Palo Alto, CA, USA).

RNA sequencing library preparation was conducted using the NEBNext Ultra RNA Library Prep Kit for Illumina by following manufacturer’s recommendations (NEB, Ipswich, MA, USA). Briefly, mRNAs were first enriched with Oligod(T) beads. Enriched mRNAs were fragmented for 15 min at 94 °C. First-strand and second-strand cDNA were subsequently synthesized. cDNA fragments were end-repaired and adenylated at 3’ends, and universal adapter was ligated to cDNA fragments, followed by index addition and library enrichment with limited cycle PCR. Sequencing libraries were validated using a DNA Chip on the Agilent 2100 Bioanalyzer (Agilent Technologies, Palo Alto, CA, USA), and quantified by using Qubit 2.0 Fluorometer (Invitrogen, Carlsbad, CA) as well as by quantitative PCR (Applied Biosystems, Carlsbad, CA, USA).

### Sequencing and preprocessing

The sequencing libraries were multiplexed and clustered on one lane of a flow cell, using the cBOT from Illumina. After clustering, the flow cell was loaded on the Illumina HiSeq 2500 instrument according to manufacturer’s instructions. The samples were sequenced using paired-end, 100 nucleotide length reads. Image analysis and base calling were conducted by the HiSeq Control Software (HCS) on the HiSeq 2500 instrument. Raw sequence data (.bcl files) generated from Illumina HiSeq 2500 were converted into fastq files and de-multiplexed using Illumina CASSAVA 1.8.2 program. One mis-match was allowed for index sequence identification. The quality of the sequencing reads was interrogated with FastQC (version 0.11.5) and found to be excellent. Reads were aligned using the STAR aligner^38^ (version 2.5.2) using basic 2-pass mapping, with all 1st pass junctions inserted into the genome indices on the fly to the GENCODE release 31 GRCh38.p12 genome using the GENCODE v31 transcript annotation, with an average of 92% of reads aligning giving an average of 24.8 M of aligned sequence per sample.

### Data analysis

RNA-seq sample identity was verified by comparison to DNA-WGS samples collected and analyzed for the same patient cohort using the BAM-matcher tool^39^ and a panel of 7550 exonic SNP sites extracted from the 1000 Genomes database.

Analysis and visualization of the resulting data were performed using R version 4.0.0 (R Foundation for Statistical Computing, Vienna, Austria). Patient cohort characteristics were assessed for differences between PD patients and HC. Age data were approximately normally distributed and were tested for significant differences using a Student t-test. Statistical differences in MDS-UPDRS (I–III & total), MoCA, and PDQ39 scores were assessed with Wilcoxon rank sum tests as the data was not normally distributed. MDS-UPDRS IV score was not tested for significance as all subjects had a score of 0. Categorical cohort data was assessed for statistical differences using Fischer’s exact tests. Aligned reads were quantified using the GenomicAlignments^40^ package (Bioconductor) with the “IntersectionStrict” setting. Differential expression analysis was performed using DESeq2^41^. Based on the degree of expression difference observed between male and female subjects, a model that incorporated case/control status, sex, and the interaction between case/control status and sex was utilized for determining differential expression between PD and HC. Contrasts between PD and HC were extracted for each sex and used in downstream analyses.

Principal component analysis (PCA) was conducted by singular value decomposition of the centered data matrix on the most 1000 variable genes in the entire dataset. The loadings of the first (x-axis) and second (y-axis) principal components were plotted.

Fold-change and significance (adjusted *p*-values) of differential gene expression between PD and HC were visualized using volcano plots for each sex. Unbiased clustering of subjects by hierarchal clustering was conducted using the WPGMA method using the top 50 differentially expressed genes between PD and HC in females and the 15 differentially expressed genes in males as input; resulting data were plotted as heatmaps for visualization.

Additionally, differential gene expression between PD and HC were used to characterize states of monocyte activation through Gene Set Enrichment Analysis^42^ (GSEA) separately in each sex. Genes were ranked by the absolute value of the Wald test statistic calculated during differential expression analysis between PD and HC for input into GSEA. Gene sets (modules) established through Weighted Gene Co-expression Network Analysis (WGCNA) by Xue et al. of a series of reference states representing differential activation of monocytes were utilized^24^. Each gene in modules of interest was plotted with the gene’s position in the ranked gene list on the x-axis and the running enrichment score on the y-axis.

GSEA was also utilized to interrogate which human Kyoto Encyclopedia of Genes and Genomes (KEGG) pathways were most impacted between PD and HC in each sex.

### Reporting summary

Further information on research design is available in the Nature Research Reporting Summary linked to this article.

## Supplementary information

Reporting Summary

## Data Availability

The subject data and biospecimens were collected as part of an NIH funded project, are deposited in the NIH *Parkinson’s Disease Biomarkers Program (PDBP) (study ID* PDBP-STUDY0000224), and are available at pdbp.ninds.nih.gov. RNA-seq data are available at dbGaP under Study Accession phs002063.v1.p1 (https://dbgap.ncbi.nlm.nih.gov).

## Code availability

All scripts used to generate and analyze data are available from the authors upon reasonable request. All versions of software utilized in data analysis are noted in the methods section.
