# Supplementary material for: Sex-based differences in the activation of peripheral blood monocytes in early Parkinson disease
Source: NPJ Parkinsons Dis. 2021 Apr 13;7:36. doi: 10.1038/s41531-021-00180-z (PMC8044127; doi:10.1038/s41531-021-00180-z)
Supplement: Supplementary file 1 — Reporting Summary [file 41531_2021_180_MOESM1_ESM.pdf]

nature research

Corresponding author(s): David Standaert

Last updated by author(s): 11/9/2020

Reporting Summary

Nature Research wishes to improve the reproducibility of the work that we publish. This form provides structure for consistency and transparency in reporting. For further information on Nature Research policies, see our Editorial Policies and the Editorial Policy Checklist.

Statistics

For all statistical analyses, confirm that the following items are present in the figure legend, table legend, main text, or Methods section.

n/a	Confirmed	
☐	☒	The exact sample size (n) for each experimental group/condition, given as a discrete number and unit of measurement
☐	☒	A statement on whether measurements were taken from distinct samples or whether the same sample was measured repeatedly
☐	☒	The statistical test(s) used AND whether they are one- or two-sided<br>*Only common tests should be described solely by name; describe more complex techniques in the Methods section.*
☐	☒	A description of all covariates tested
☐	☒	A description of any assumptions or corrections, such as tests of normality and adjustment for multiple comparisons
☐	☒	A full description of the statistical parameters including central tendency (e.g. means) or other basic estimates (e.g. regression coefficient) AND variation (e.g. standard deviation) or associated estimates of uncertainty (e.g. confidence intervals)
☒	☐	For null hypothesis testing, the test statistic (e.g. F, t, r) with confidence intervals, effect sizes, degrees of freedom and P value noted<br>*Give P values as exact values whenever suitable.*
☒	☐	For Bayesian analysis, information on the choice of priors and Markov chain Monte Carlo settings
☒	☐	For hierarchical and complex designs, identification of the appropriate level for tests and full reporting of outcomes
☒	☐	Estimates of effect sizes (e.g. Cohen's d, Pearson's r), indicating how they were calculated

Our web collection on statistics for biologists contains articles on many of the points above.

Software and code

Policy information about availability of computer code

Data collection	RNA-sequencing: HiSeq Control Software (HCS), Illumina CASSAVA 1.8.2
Data analysis	RNA-sequencing: FastQC version 0.11.5, STAR aligner version 2.5.2, R version 3.5.3 & 4.0.0, R packages: GenomicAlignments version 1.18.1, DESeq2 version 1.22.2, fgsea version 1.14.0

For manuscripts utilizing custom algorithms or software that are central to the research but not yet described in published literature, software must be made available to editors and reviewers. We strongly encourage code deposition in a community repository (e.g. GitHub). See the Nature Research guidelines for submitting code & software for further information.

Data

Policy information about availability of data

All manuscripts must include a data availability statement. This statement should provide the following information, where applicable:

- Accession codes, unique identifiers, or web links for publicly available datasets
- A list of figures that have associated raw data
- A description of any restrictions on data availability

The subject data and biospecimens were collected as part of an NIH funded project, are deposited in the NIH Parkinson's Disease Biomarkers Program (PDBP) (study ID PDBP-STUDY0000224), and are available at pdbp.ninds.nih.gov. RNA-seq data are available at dbGaP under Study Accession phs002063.v1.p1 (https://dbgap.ncbi.nlm.nih.gov).

Field-specific reporting

Please select the one below that is the best fit for your research. If you are not sure, read the appropriate sections before making your selection.

[x] Life sciences [ ] Behavioural & social sciences [ ] Ecological, evolutionary & environmental sciences

For a reference copy of the document with all sections, see nature.com/documents/nr-reporting-summary-flat.pdf

Life sciences study design

All studies must disclose on these points even when the disclosure is negative.

Sample size	The study population was recruited as part of the Alabama Udall Exploratory Center, NIH grant P20 NS092530. The sample was powered to detect differences in microRNA expression between PD and control. We did not a priori calculate the power for the sequencing experiments reported here because insufficient data were available to estimate power for this approach.
Data exclusions	A total of 40 patients were recruited to the study. Six patients had inadequate samples or sequencing that failed quality control. The remaining 34 are reported.
Replication	We have not performed replication. This will require recruitment of a new and larger cohort of patients. The results of the present study, however, are critical to the design of such future replication cohorts.
Randomization	Participants were not randomly assigned to groups as we studied the difference between a disease state (Parkinson disease) and healthy controls.
Blinding	Processing of the biospecimens, including separation of monocytes and preparation for RNA seq analysis, were conducted by personnel blinded to the diagnosis.

Reporting for specific materials, systems and methods

We require information from authors about some types of materials, experimental systems and methods used in many studies. Here, indicate whether each material, system or method listed is relevant to your study. If you are not sure if a list item applies to your research, read the appropriate section before selecting a response.

Materials & experimental systems

n/a	Involved in the study	
[x]	[ ]	Antibodies
[x]	[ ]	Eukaryotic cell lines
[x]	[ ]	Palaeontology and archaeology
[x]	[ ]	Animals and other organisms
[ ]	[x]	Human research participants
[x]	[ ]	Clinical data
[x]	[ ]	Dual use research of concern

Methods

n/a	Involved in the study	
[x]	[ ]	ChIP-seq
[ ]	[x]	Flow cytometry
[x]	[ ]	MRI-based neuroimaging

Human research participants

Policy information about studies involving human research participants

Population characteristics	Evidence of a difference between PD and HC in the proportion of males, proportion of Caucasian race, and age of patients (at blood collection visit) were not observed. However, family history of PD, Movement Disorder Society-Unified Parkinson's Disease Rating Scale (MDS-UPDRS; I-III & total) scores, Montreal Cognitive Assessment (MoCA) score, and Parkinson's Disease Questionairre-39 (PDQ39) score were significantly different between cases and HC (p< 0.05 for all). Of note is that no patient within the cohort scored any points on the MDS-UPDRS IV, a scale measuring symptoms of later stage PD, therefore significance was not tested. Additionally, there were no significant differences in any of the subject cohort characteristics, including age, between males and females.
Recruitment	Patients with early PD were diagnosed using UK Brain Bank criteria (bradykinesia and either: 4-6 Hz resting tremor or rigidity). We included male or female subjects that were within 2 years of diagnosis, who were age 30 years or older at time of PD diagnosis, and who were Hoehn and Yahr stage I-II at the time of study entry. All PD subjects had no history of prior treatment with PD medications. Other exclusion criteria for PD subjects included clinical suspicions of atypical PD syndromes. Healthy controls were over 30 years old, had no current diagnosis of PD or other neurodegenerative disorder, no history of PD in first-degree blood relatives, and scored positive on no more than three items on the PD Screening Questionnaire.
Ethics oversight	This study was reviewed and approved by the University of Alabama at Birmingham (UAB) Institutional Review Board. All subjects signed written informed consent to participate in this study.

Note that full information on the approval of the study protocol must also be provided in the manuscript.

Flow Cytometry

Plots

Confirm that:

- [x] The axis labels state the marker and fluorochrome used (e.g. CD4-FITC).
- [x] The axis scales are clearly visible. Include numbers along axes only for bottom left plot of group (a 'group' is an analysis of identical markers).
- [x] All plots are contour plots with outliers or pseudocolor plots.
- [x] A numerical value for number of cells or percentage (with statistics) is provided.

Methodology

Sample preparation	Up to 50 ml of venous blood was collected from each participant in ethylenediamine-tetraacetic acid tubes and peripheral blood mononuclear cell (PBMCs) isolation. PBMCs were isolated from whole blood by Ficoll-PaqueTMdensity-gradient centrifugation, and then divided into aliquots for monocyte analysis and RNA extration. All samples were processed within 2 h of receipt. Monocyte population was analyzed using PBMCs (1 x 106), which were blocked with Human TruStain FcX (BioLegend, San Diego, CA) prior to labeling with fluorochrome-conjugated monoclonal antibodies to monocytes, neutrophils, NK cells, T-cell and B-cell surface markers. Antibodies were from Biolegend except where noted otherwise: anti-CD45 Pacific Blue (clone HI30); anti-CD3 Brilliant Violet 605/Brilliant Violet 650 (clone OKT3); anti-CD14 FITC (clone HCD14); anti-CD16 APC (clone 3G8); anti-CD19 Brilliant Violet 650 (clone HIB19); anti-CD66b PE (clone G10F5) and BUV395 anti-CD56 (clone NCAM16.2, BDBioscience). All antibodies were diluted to 1:200 and incubation time on ice was 30-45 min. Cells were fixed with 2% paraformaldehyde. The Aqua Live/Dead Kit was used to assess cell viability (ThermoFisher Scientific).
Instrument	Flow cytometry was performed on an LSRII flow cytometer (BD Biosciences).
Software	Flow cytometry data analyzed using FlowJo software (Tree Star, Inc, Ashland, OR). Statistical analyses were done using descriptive statistics and Student's t test distribution and one-sided two-sample Mann-Whitney rank-sum test comparing percentages of monocyte subsets. (as a percentage of CD45+CD3+ lymphocytes) and B-cell subsets (CD19+CD3- lymphocytes) between PD patients and HC. Immune cells were gated using forward scatter and side scatter, and single cells were identified using forward-scatter area versus width.
Cell population abundance	N/A
Gating strategy	PBMCs were stained with antibodies to CD45, CD3, CD14, CD16, CD19, CD56, and CD66b, and then single CD45+ live cells were gated as CD56-CD66b-CD3-CD19- monocytes. As the next step, monocyte subsets were determined using CD14 and CD16, evaluating Classical (CD14++CD16-), Intermediate (CD14++CD16+) and Non-classical (CD14+CD16++) monocytes.

- [x] Tick this box to confirm that a figure exemplifying the gating strategy is provided in the Supplementary Information.
